# Early detection of heart failure using in-patient longitudinal electronic health records

**DOI:** 10.1371/journal.pone.0314145

**Published:** 2024-12-18

**Authors:** Ignat Drozdov, Benjamin Szubert, Clare Murphy, Katriona Brooksbank, David J. Lowe

**Affiliations:** 1 Bering Limited, London, United Kingdom; 2 Cardiology, Royal Alexandra Hospital, Paisley, United Kingdom; 3 Institute of Cardiovascular and Medical Sciences, BHF Glasgow Cardiovascular Research Centre, University of Glasgow, Glasgow, United Kingdom; 4 Emergency Department, Queen Elizabeth University Hospital, Glasgow, United Kingdom; 5 Institute of Health and Wellbeing, University of Glasgow, Glasgow, United Kingdom; KPC Medical College and Hospital, INDIA

## Abstract

Heart Failure (HF) is common, with worldwide prevalence of 1%-3% and a lifetime risk of 20% for individuals 40 years or older. Despite its considerable health economic burden, techniques for early detection of HF in the general population are sparse. In this work we tested the hypothesis that a simple Transformer neural network, trained on comprehensive collection of secondary care data across the general population, can be used to prospectively (three-year predictive window) identify patients at an increased risk of first hospitalisation due to HF (HHF). The model was trained using routinely-collected, secondary care health data, including patient demographics, A&E attendances, hospitalisations, outpatient data, medications, blood tests, and vital sign measurements obtained across five years of longitudinal electronic health records (EHRs). The training cohort consisted of n = 183,894 individuals (n = 161,658 age/sex-matched controls and n = 22,236 of first hospitalisation due to HF after a three-year predictive window). Model performance was validated in an independent testing set of n = 8,977 patients (n = 945 HHF patients). Testing set probabilities were well-calibrated and achieved good discriminatory power with Area Under Receiver Operating Characteristic Curve (AUROC]) of 0.86, sensitivity of 36.4% (95% CI: 33.33%-39.56%), specificity of 98.26% (95% CI: 97.95%-98.53%), and PPV of 69.88% (95% CI: 65.86%-73.62%). At Probability of HHF ≥ 90% the model achieved 100% PPV (95% CI: 96.73%-100%) and sensitivity of 11.7% (95% CI: 9.72%-13.91%). Performance was not affected by patient sex or socioeconomic deprivation deciles. Performance was significantly better in Asian, Black, and Mixed ethnicities (AUROC 0.932–0.945) and in the 79–86 age group (AUROC 0.889). We present the first evidence that routinely collected secondary care health record data can be used in the general population to stratify patients at risk of first HHF.

## Introduction

Heart Failure (HF) is a multi-faceted clinical syndrome characterised by the reduced ability of the heart to pump and/or fill with blood [1]. HF has been defined as a global pandemic [2], with prevalence of 1%-3% worldwide [1]. The lifetime risk of developing HF for individuals 40 years or older is 20% [3], whilst one-, five-, and 10-year survival rates have remained largely unchanged at 80.8%, 48.2%, and 26.2% respectively [4]. Health economic impacts are considerable, with the total cost of HF in USA and UK estimated to be $30.7 billion and £2 billion respectively, with projections suggesting an increase in costs by 127% 2030 [3,5,6].

Historically, post-diagnostic care has been the focus of HF patient management and relatively little attention has been devoted to earlier detection and diagnosis of HF [7–9]. Indeed, for at least 68% of all new cases of HF [10], the diagnosis has been made during an acute hospitalisation, yet half of these people had symptoms for up to 5 years prior to hospital admission [11]. There is an urgent need to embed effective strategies within HF services and primary care, to enable earlier disease detection and evidence-based treatment initiation, to reduce the risk of unplanned hospitalisation and premature mortality.

The increased availability of electronic health records (EHRs) opens new opportunities to develop predictive case finding algorithms that facilitate early HF detection and surveillance [11]. However, despite proliferation of machine-readable datasets, development and scaling of predictive models has been limited [12]. Complexities of real-world clinical data, replete with thousands of potential predictor variables and missing values are seen as the key barriers to implementation [12–14]. Deep Neural Networks (DNNs) have emerged as robust tools with applications to sequence prediction within mixed modality data sets [13–16]. The key advantages of DNN methods are their ability to handle large volumes of relatively noisy data, including errors in labels, as well as large numbers of input variables [14].

Existing HF hospitalisation prediction models have been developed in groups of patients who are already at an increased risk of adverse outcomes, including patients who: were undergoing clinically indicated cardiovascular magnetic resonance imaging (cMRI) [17]; had previous hospitalisations for HF [18]; have undergone cardiac transplantation [19]; or have an increased risk of HF-related mortality [20]. Furthermore, although longitudinal EHRs combined with conventional machine learning techniques (Logistic Regression and Random Forest) have shown some utility in incident HF prediction [8], model performance was marginal, with Area Under Received Operating Characteristic Curve (AUROC) of 0.80 for prediction windows ≤1 year, declining rapidly for prediction window lengths longer than 2 years [8].

In this work we tested the hypothesis that a simple Transformer neural network, trained on comprehensive collection of secondary care data in a general population, including inpatient and outpatient interactions, can be used effectively to predict patients at an increased risk of first hospitalisation with HF (HHF).

## Materials and methods

Delegated research and ethics approvals for this study were granted by the Local Advisory Committee at NHS Greater Glasgow and Clyde (NHS GG&C). Cohorts and de-identified linked data were prepared by the West of Scotland Safe Haven at NHS GG&C. In Scotland, patient consent is not required where routinely collected patient data are used for research purposes through an approved Safe Haven [21]. For that reason, informed consent was not required and was not sought. All research was performed in accordance with relevant guidelines/regulations.

### Study population

A nested case-control design was used on a large patient population from NHS GG&C collected between 2002 and 2021. The data was accessed and analysed between December 12, 2022 and January 4, 2024. The authors did not have access to information that could identify individual participants during or after data collection. Qualifying criteria for incident 1st HHF was adopted using the International Classification of Diseases–Tenth Revision (ICD-10) codes for HF (I11.0, I13.0, I13.2, I25.5, I42.0, I42.9, I50.0, I50.1, I50.9, I50.90, I50.91), in the first coding position. All incident 1st HHF cases had at least 60 months before the first occurrence of a HF ICD-10 code diagnosis, without an indication that HF was previously diagnosed or treated. Finally, the incident first HHF cohort comprised of patients still alive 12 months following the first occurrence of HF ICD-10 code diagnosis. All patients had to have at least 60 months of in-patient clinical history. Exclusion criteria consisted of patients younger than 40 years of age at prediction date.

Up to ten eligible sex- and age-matched controls were selected for each incident HHF case.

Dataset was split into randomised training (90%), validation (5%), and testing (5%) partitions, such that distribution of age, gender, and outcomes were stratified across each partition. To avoid data leakage across data partitions, we ensured that there were no overlapping patient identifiers.

### Data representation and ground truthing

**Fig 1:**
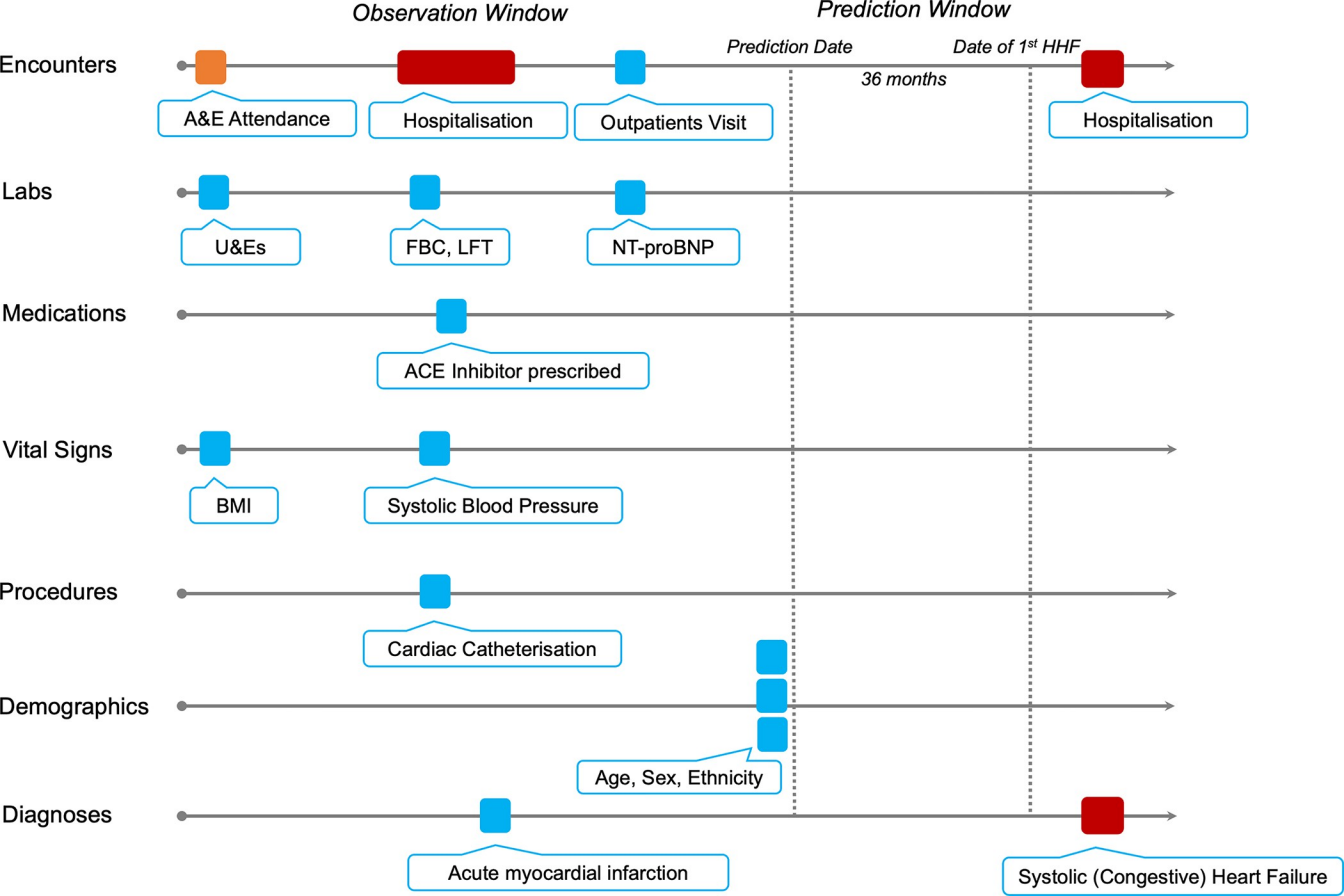
Schematic representation of an EHR vector. The patient’s timeline is represented by horizontal arrows and each data point is depicted by colour-coded tokens. Predictive models were trained on the data in the Observation Window, whilst a binary outcome for first HHF was used as a ground truth.

Each patient’s longitudinal EHR vector was split into an Observation and Prediction Windows (**Fig 1**). Prediction Date for case patients was calculated as the date 36 months prior to the first hospitalisation with one or more HF-related ICD10 codes (see Study Population section). Prediction Date for age- and sex-matched controls was calculated as the date 36 months prior to the last EHR entry. Observation Window comprised all EHR vectors during a five-year period in the run up to the Prediction Date. Only data in the observation window was used to represent the patient during model training, validation, and testing.

Patient features used in predictive modelling are shown in **Table 1**. For each patient, two feature vector representations were generated. The first representation consisted of static features–age, ethnicity and sex. The second representation reflected dynamic features associated with inpatient and outpatient activity over a five-year period of the Observation Window. This temporal input vector was discretised into twelve exponentially increasing time bins, such that the most recent time points were assigned to the shortest time bin. This is expressed as T * (1-np.linspace(0.0, 1.0, n = 12)**2), where T is 60 months (5-year prediction window) and n is the number of bins. If a feature (e.g. ICD-10 codes) within an observation window contained multiple values, the most frequent value was retained. In cases where numerical feature (e.g. BMI) contained multiple entries within one observation window, an average was calculated. Missing values were filled by forward propagation.

**Table 1:** Features of the patient EHR used as inputs into the predictive model.

Data Type	Description	Percentage missing information
Age	Patient age at Prediction Date	0%
Sex	Patient sex at Prediction Date	0%
Ethnicity	Patient ethnicity at Prediction Date	8%
Medications	British National Formulary (BNF) Subsection codes	<5%
Laboratories	Urea, Estimated Glomerular Filtration Rate (EGFR), Creatinine, Potassium, Haemoglobin, Neutrophils, Lymphocytes, N-terminal pro b-type natriuretic peptide (NT-proBNP),	<5
Hospitalisations	All ICD-10 codes associated with admission, Length of stay associated with admission, Primary clinical speciality	0%
A&E Attendances	ICD-10 codes associated with attendances	0%
Outpatients	Appointment speciality, ICD-10 codes associated with each appointment	0%
Vital Signs	Systolic blood pressure, Body Mass Index (BMI)	58%

Numerical data was scaled to a range between 0 and 1, whilst categorical data was represented as 32-dimensional vectors of a large pre-trained language model [22,23]. Briefly, an uncased DistilBERT model was initialised using weights provided by Sanh et al [24]. We then continued to pre-train the model for three epochs using n  =  2,067,531 full text PubMed articles distributed under Creative Commons (CC) BY or CC0 license [25], totalling n  =  224,427,218 sentences. All words were converted to lower case and punctuation was removed. Tokenization was performed using a WordPiece tokenizer with a vocabulary size of 52,000 words and word occurrence frequency of greater or equal to two. Embedding layer was modified to 32 dimensions and the 32 dimensional vector was used to represent all categorical variables, including sex and ethnicity.

### Model training

Given significant variation in length and density of patient records (e.g., vital sign measurements in an intensive care unit vs outpatient clinic), we formulated a simple Transformer architecture with multi-head attention [26], to take advantage of such data.

Input layers of the Transformer network were adjusted to concurrently use time-invariant and time-dependent features. Multiple inputs were concatenated along a horizontal axis and passed to four Transformer encoder blocks with multi-head attention. Four attention heads were used with head size fixed at 256. The classification head of the network consisted of a global average pooling layer, followed by a dense layer with rectified linear unit [27] activation and a dropout layer. A softmax activation function was applied to the final dense layer.

The number of neurons in the penultimate dense layer and the dropout rate were tunable hyperparameters optimized during training using the Hyperband algorithm [28], with the best set of parameters corresponding to the lowest sparse categorical cross-entropy loss on the validation set. The number of neurons was selected from the range of [32, 512], and the dropout rate took values from the range [0, 0.2].

Training was performed with batch size of 512 using an Adam optimizer with a learning rate of 1 × 10^−4^ while minimizing the sparse categorical cross-entropy loss.

The network was trained to output the probability of first HHF following a 36-month prediction window. Training was terminated early if validation loss did not improve after ten consecutive epochs.

### Statistical analysis

Model performance was assessed using area under the receiver operating characteristic (AUC) curve, overall accuracy, sensitivity, specificity, and positive predictive value (PPV). For AUC measures, 95% CIs were calculated empirically using 2,000 bootstrap samples. CIs for sensitivity, specificity and accuracy are exact Clopper-Pearson CIs. Patient demographics were compared across the training/validation, internal testing, and clinical evaluation sets using ANOVA for continuous variables and Chi-square for categorical variables. P-values < 0.05 were considered as significant.

Dimensionality reduction was performed out using the Ivis algorithm [29]. Briefly, prior to analysis, categorical variables were one-hot encoded, whilst numerical variables were scaled to values between 0 and 1. The dataset was reduced to two components using the ‘maaten’ twin Neural network architecture and default Ivis hyperparameter values. To identify the salient features captured by the Transformer model, we calculated the coefficient of determination (*R*^2^) between low-dimensional representations of the model global average pooling layer and training set features. Where categorical features were used, their numerical representation were extracted from the model’s feature embedding layer.

All statistical tests were carried out using the SciPy module (version 1.7.3) for Python (version 3.9.14).

## Results

### Dataset characteristics

**Fig 2:**
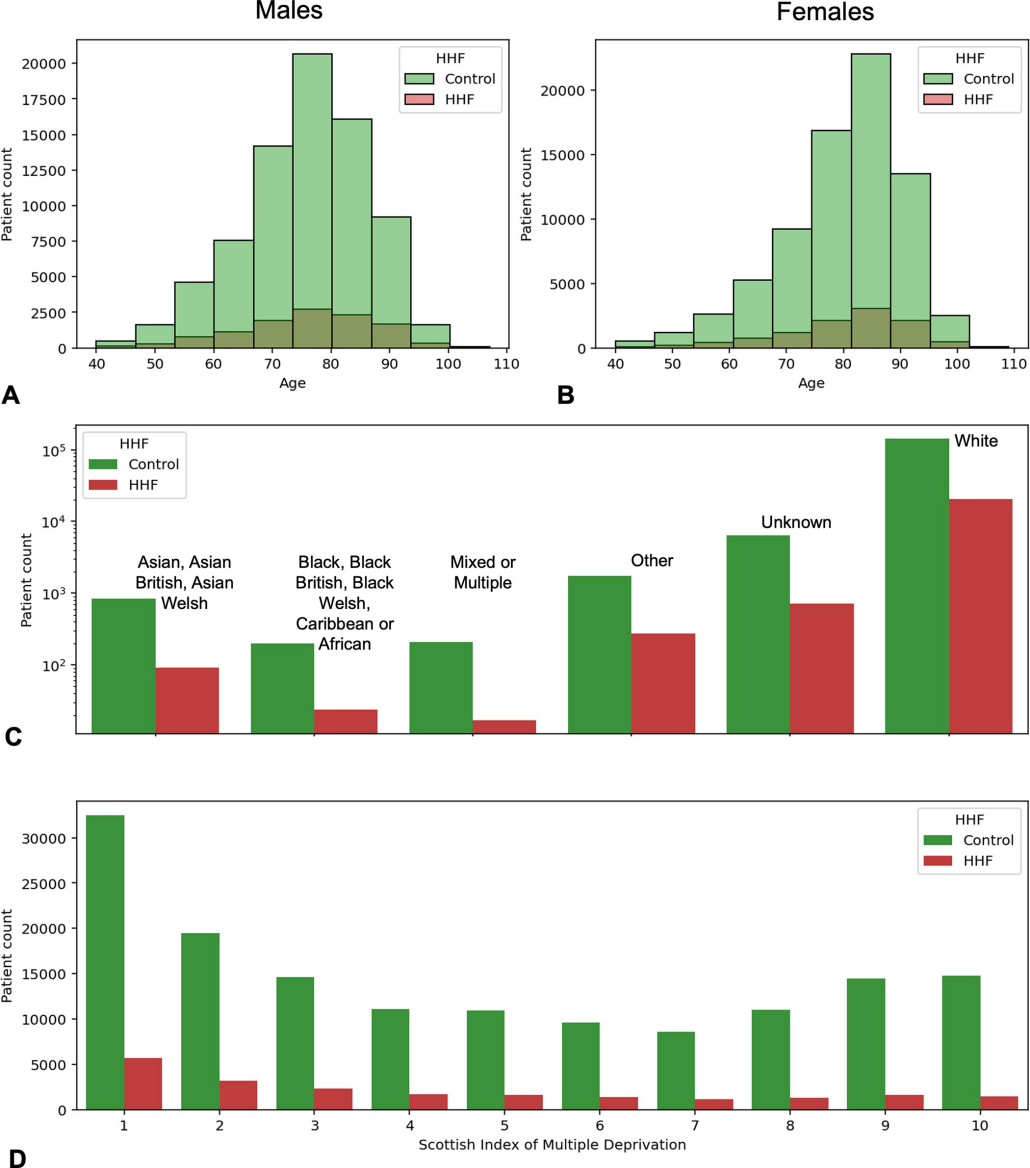
Demographic characteristics of the dataset. **A, B)** Histograms of patient age distributions at Prediction Date. Green and Red bars reflect patients without and with first HHF. **C)** Bar plots demonstrating incidence of first HHF (red) and Controls (green) across broad ethnicity groupings. **D)** Bar demonstrating incidence of first HHF (red) and Controls (green) across Scottish Index of Multiple Deprivation (SIMD) deciles. Patients in the most and least deprived 10% of the population have SIMDs of one and ten respectively.

Electronic Health Records, collected consecutively over 19 years, from n = 310,859 patients across Greater Glasgow and Clyde were included in initial data extract. Following application of the inclusion and exclusion criteria, the final cohort consisted of n = 183,894 individuals (n = 161,658 controls and n = 22,236 cases). The average age for male and female patients with first HHF was 75.7 (+/- 11.3) and 80.4 (+/-11.4) years respectively. Similarly, the average age for male and female controls was 75.7 (+/- 10.4) and 80.1 (+/- 10.8) years respectively (**Fig 2A and 2B**). There was no statistically-significant difference between age distribution of cases and controls (Kolmogorov-Smirnov p-value = 0.86).

The most and least common patient ethnicities in the dataset were White (94%) and Black (Black British, Black Welsh, Caribbean or African [0.13%], **Fig 2C**). Incidence of first HHF across all ethnicity groups ranged from 8% (Mixed of Multiple) to 15.5% (Other). First HHF was most prevalent (17.5%) in the first decile of the Scottish Index of Multiple Deprivation (SIMD) (**Fig 2D**).

### Prediction of First HHF

**Fig 3:**
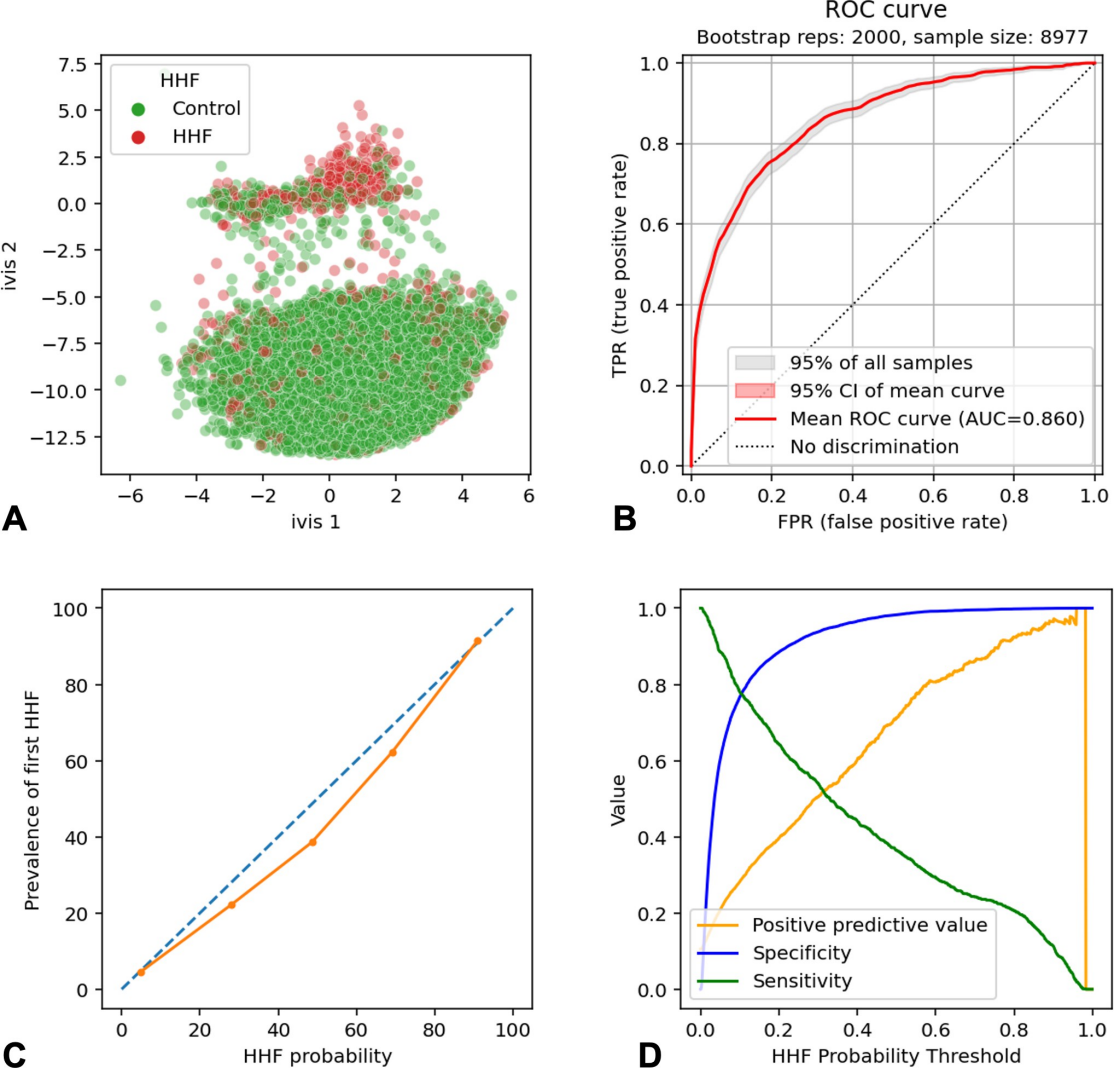
Transformer neural network performance in detection of testing set patients (n = 8,977) with first HHF after a 3-year predictive window. **A)** Scatterplot shows two-dimensional twin neural network (Ivis) embedding of the global average pooling layer values in the trained Transformer neural network. Each point represents a single patient in the testing set. Green and red dots represent controls and first HHF cases respectively. **B)** Received Operating Characteristic Curve for first HHF prediction. Shaded areas are 95% Confidence Intervals (CIs) generated using 2000 bootstrapped samples. **C)** Calibration plot demonstrating the relationship between model probabilities (HHF probability) and prevalence of first HHF in the testing set. D) Line plots of model positive predictive value (orange), specificity (blue), and sensitivity (green) at each HHF probability threshold. AUC = Area Under the Curve.

Transformer neural network was trained for three epochs before reaching early stopping criteria. Dimensionality reduction of the global average pooling layer confirmed model propensity to learn the target class (**Fig 3A**). The model performance was evaluated on an independent set of n = 8,977 patients (n = 945 patients with first HHF). The model achieved AUROC of 0.86 (95% CI: 0.860, 0.861) (**Fig 3B**). Binarizing predicted cases and controls using an operating point of Probability of HHF ≥ 50%, resulted in sensitivity of 36.4% (95% CI: 33.33%-39.56%) specificity of 98.26% (95% CI: 97.95%-98.53%), and PPV of 69.88% (95% CI: 65.86%-73.62%). Model probabilities were well calibrated, with a Pearson’s R^2^-values of 0.99 (two-sided p-value = 9.9x10^-4^, **Fig 3C**). At Probability of HHF ≥ 90% the model achieved 100% PPV (95% CI: 96.73%-100%) and sensitivity of 11.7% (95% CI: 9.72%-13.91%, **Fig 3D**).

**Fig 4:**
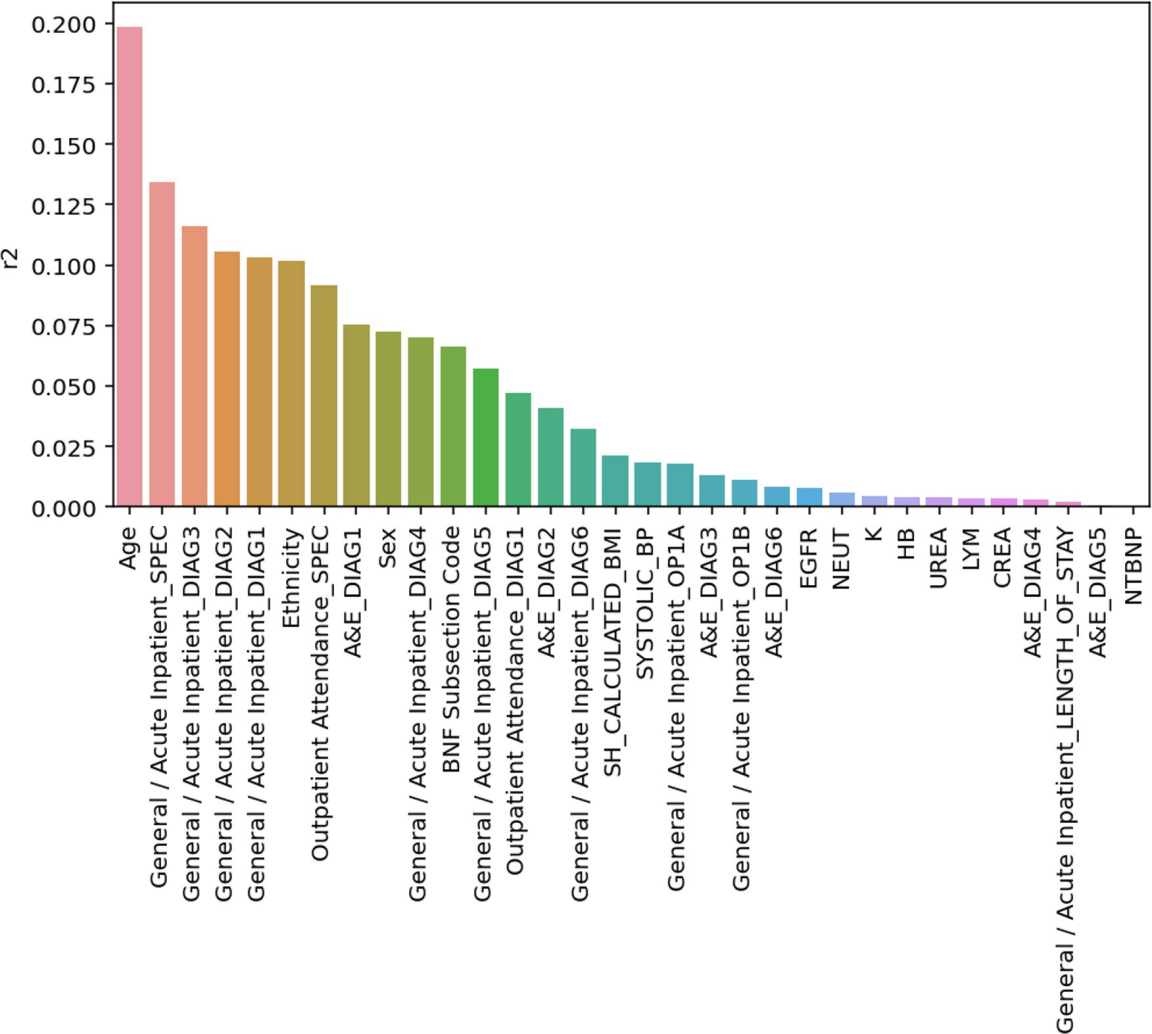
Bar plot showing model input features and their respective coefficient of determination (R^2^) values. Values reflect variance within the global average pooling layer explained by each feature.

Coefficients of Determination (see **Methods**) were calculated for every input feature. Features that correlated the most with the global average pooling layer of the model were Age (R^2^ = 0.198), Clinical Specialty assigned to the patient at the time of an in-patient admission (R^2^ = 0.134), and diagnoses following in-patient stay (R^2^ = 0.103–0.115). Blood tests, including NTproBNP were expressed low Coefficients of Determination (R^2^ = 0–0.008) (**Fig 4**).

Model misclassifications were interpretable. For example, at probability of HHF ≥ 50%, the model identified n = 140 false positive cases. Of these, n = 16 patients (11%) and n = 12 (8.5%) had recent diagnoses (within three years) of Atherosclerotic heart disease of native coronary artery (I25.1) and Unstable Angina (I20.0) respectively. Furthermore, n = 32 (22.8%), n = 30 (21.4%), and n = 29 (20.7%) patients had at least a three-year history of lipid regulators, beta adrenoreceptor blockers, and antiplatelet drug usage. Finally, n = 127 patients (90.7%) had in-patient stays under Cardiology services as their primary specialty.

Conversely, at probability of HHF ≥ 50%, the model identified n = 601 false negative cases. Of these, the most common diagnoses upon discharge over a five-year Observation Window were Unspecified Cataract (n = 73 patients [12.1%], H26.9), Unspecified Chest Pain (n = 35 patients [5.8%], R07.4), and Diverticular disease of large intestine without perforation or abscess (n = 26 patients [4.3%], K57.3). Interestingly, only n = 29 patients (4.8%) had a diagnostic code pertaining to the cardiovascular system. The most common primary specialty amongst the false negative cases was Opthalmology (n = 511 patients, 85%), followed by Trauma and Orthopaedics (n = 348 patients, 57.9%) and General Surgery (n = 319 patients, 53%). N = 276 patients (45.9%) were under Cardiologist management. Finally, the most frequently prescribed medication classes were Non-opioid analgesics (n = 326 patients, 54.2%), Renin-Angiotensin system drugs (n = 292 patients, 48.6%), and lipid regulating drugs (n = 275 patients, 45.8%). Of note, whilst only one patient had an acute myocardial infarction within the Observation Window, n = 246 (40.9%) experienced a myocardial infarction within the Prediction Window period.

### Subgroup analysis

Model performance was assessed in patient subgroups, defined by sex, age quartiles, ethnicity, and SIMD quartiles (**Table 2**). Model AUROC was consistent across Male and Female patients. The performance in the third age quartile (79–86 years old) was significantly better compared to the rest of the population (AUROC:0.889 vs. 0.840–0.859, DeLong’s p-values: 0.03–0.05). The model also performed better in patients of Asian, Black, and Mixed ethnicities (AUROC: 0.932–0.945 vs. AUROC: 0.859–0.880, DeLong’s p-values: 0.001–0.03), with positive predictive values of 100% at probability of first HHF ≥50%. There was no statistically significant difference in performance across SIMD quartiles.

**Table 2:** Model performance metrics across confounder subgroups using operating point of ≥50% (probability of first HHF). 95% CIs are shown in parentheses. AUROC = Area under Receiver Operating Characteristics Curve.

Confounder		Number of Patients	AUROC	Sensitivity	Specificity	Positive Predictive Value
**Sex**	Male	4,510	0.861 (0.859–0.861)	36% (35%-38%)	99% (97%-100%)	75% (73%-79%)
	Female	4,467	0.855 (0.855–0.857)	37% (35%-39%)	98% (97%-100%)	68% (66%-71%)
**Age**	40–71	2,182	0.852 (0.852–0.853)	39% (37%-41%)	97% (96%-100%)	65% (64–67%)
	71–79	2,170	0.859 (0.858–0.860)	43% (41%-44%)	98% (97%-100%)	68% (66%-70%)
	79–86	2,248	0.889 (0.888–0.889)	41% (40%-43%)	99% (97%-100%)	79% (78%-82%)
	86+	2,377	0.84 (0.83–0.841)	24% (23%-27%)	99% (97%-100%)	77% (76%-79%)
**Ethnicity**	Asian, Asian British, Asian Welsh	140	0.940 (0.940–0.944)	17% (15%-19%)	100% (97%-100%)	100% (98%-100%)
	Black, Black British, Black Welsh, Caribbean or African	113	0.932	18% (16%-21%)	100% (96%-100%)	100% (97%-100%)
	Mixed or Multiple	120	0.945 (0.943–0.948)	21% (19%-24%)	100% (98%-100%)	100% (98%-100%)
	White	8,151	0.859 (0.859–0.856)	38% (37%-39%)	98% (97%-100%)	71% (70%-73%)
	Other	114	0.874 (0.869–0.873)	29% (28%-31%)	99% (98%-100%)	80% (79%-82%)
	Unknown	339	0.880 (0.880–0.885)	13% (12%-16%)	100% (97%-100%)	100% (98%-100%)
**SIMD**	1–2	2,945	0.863 (0.863–0.864)	40%	98%	75% (74–77%)
	2–4	2,164	0.852 (0.851–0.853)	32%	99%	75% (74%-77%)
	4–8	2,291	0.857 (0.857–0.858)	35%	98%	68% (67%-70%)
	8–10	1,577	0.849 (0.849–0.851)	36%	97%	64% (63%-66%)

## Discussion

HF is a considerable global health economic burden. However, techniques for early prediction and detection of HHF in the general population are sparse. We demonstrate that a simple Transformer neural network model, trained on routinely-collected secondary care electronic health record data, produces well-calibrated probabilities and achieved good discriminatory power within a long (3-year) predictive window, with AUROC of 0.86, sensitivity of 36.4% (95% CI: 33.33%-39.56%), specificity of 98.26% (95% CI: 97.95%-98.53%), and PPV of 69.88% (95% CI: 65.86%-73.62%).

The HHF classifier is a Transformer neural network, described in [26]. Traditionally, the Transformer architecture was extensively applied to natural language processing, achieving state-of-the-art performance in text annotation [22], named entity recognition [30], and representation learning [31]. More recently, the utility of the Transformer architecture was explored in longitudinal EHRs, demonstrating striking capacity to parse heterogeneous data sequences and predict multiple clinical trajectories [32,33], considerably outperforming conventional machine learning techniques. The propensity of this technique to handle large volumes of relatively noisy data, including errors in labels, as well as large numbers of input variables [14], makes it an attractive tool for interrogation of real-world EHRs.

Previous models detected incident HHF with varying degrees of accuracies, which depended heavily on the length of the prediction window (i.e. the duration of time before HF diagnosis) [34]. For example, interrogation of longitudinal EHRs with classical machine learning methods, such as Random Forests and Logistic Regression, yielded AUROC of 0.80 for prediction windows ≤1 year. This metric declined rapidly for prediction window lengths longer than 2 years (AUROC 0.58–0.74) [8]. Incident HF detection within a short predictive window (≤1 year), was improved by a Transformer neural network, trained on linked primary and secondary care EHRs from 100,071 patients in England, achieving AUROC 0.93 in 6-month incident HF detection [35].

Recently, A Cox proportional hazards model, developed to predict risk of hospitalisation for heart failure or all-cause mortality at 3 years after cardiac magnetic resonance imaging (cMRI), achieved Harrell’s C-index of 0.805 (95% CI 0.793–0.829) in the development cohort and 0.793 (0·766–0·820) in the external validation cohort [17]. Similarly, Khan and colleagues have derived race-specific and sex-specific models for the 10-year risk of incident heart failure in the general population using age, blood pressure, fasting glucose, body-mass index, cholesterol, smoking status, and QRS complex duration [36]. External validation of the Cox regression model demonstrated good discrimination (C-statistic ranging from 0.71–0.85) and strong calibration. In this work we achieve a good balance between prediction window length (3-years) and model performance (AUROC 0.86). Indeed, shorter prediction windows provide limited therapeutic benefit, with underlying disease mechanisms becoming less modifiable [17], whilst longer prediction windows may result in large number of false positives, rendering proactive therapeutic or lifestyle intervention less practicable [8,37].

The need to shift HF diagnosis upstream to improve clinical outcomes and operational efficiencies has been well-documented [38–40]. Nevertheless, the feasibility of clinical implementation of existing risk models in routine clinical practice is challenged by sparse and inconsistent availability of features such as systolic blood pressure, body mass index, and total cholesterol in asymptomatic community-dwelling population [39]. Our models were trained on opportunistically acquired data in secondary care and alleviate the need for targeted data collection in the community. We envisage a potential clinical case for a proactive community-based stratification of asymptomatic adults who may be at risk first HHF using routinely collected secondary care data.

Model performance remained consisted across patient sex and SIMD groupings. Interestingly, we observed statistically-significant improvement in model performance for patients with Asian, Black, and Mixed ethnicities (**Table 2**). These results seem to be consistent with previous findings [36], where higher C-statistic values were observed in black men and women at a 10-year risk of HF. Although it is of note that positive predictive values were 100% across these ethnicities, it is likely that observed performance improvement is simply an artifact of relatively small numbers of these patients in our testing set (n = 8,151 White patients vs. n = 353 Asian, Black, and Mixed ethnicity patients).

We utilised a data-driven strategy to delineate the salient features captured by our model by computing the coefficient of determination (*R*^2^) between low-dimensional representations of the model global average pooling layer and input features. Although a number of algorithms exist to explain black box models [41,42], they are limited to lower-dimensional tabular data. Our approach, validated in medical imaging [43], attempts to explain features captured within the unstructured temporal information. Patient age as well as primary inpatient specialty, and diagnostic codes accounted for 20%, 13%, and 11% of variance in the global average pooling embedding. Of note, natriuretic peptide concentrations had no effect on model performance. This is in contrast to a limitless-arity multiple-testing procedure approach which identified that a cumulative combination of proton pump inhibitors, high plasma BNP levels, diuretics use, advanced age, and lack of anti-dyslipidemia drugs increases the overall risk of HF [44]. This discrepancy is likely due to low clinical suspicion of HF in our training population and largely normal levels of this test in the training set.

Surprisingly, known risk factors for HF, such as high systolic blood pressure and increased BMI accounted for <3% of variance learned by the transfomer model. It is likely that this reflects poor record of these values in the secondary care EHR. For example, BMI was not recorded in 58% of the study population and in remaining patients recorded only once during a five-year observation window. Similarly, blood pressure was recorded opportunistically during an A&E attendance only in 27% of the population, with remaining values missing. It is likely that more accurate inclusion of these variables from primary care sources will both improve overall model performance and provide a realistic estimate of their contribution to patient’s risk. Nevertheless, robustness of our Transformer model to incomplete information was confirmed.

Our Transformer model presents several advantages. Firstly, it was trained on a large and diverse population using routinely-collected EHR data. Indeed, to the best of our knowledge, this is the largest training cohort to date (n = 183,894 individuals, n = 22,236 first HHF cases). This minimises selection bias and offers a robust inclusion criterion for a population-level risk stratification algorithm. Secondly, model probabilities were well calibrated and robust across, sex, ethnicities, age, and socioeconomic groups. Finally, an accurate inference at three-year prediction window resolution offers an opportunity for a timely, low-cost preventative intervention in the general population.

Our study had limitations. First, our inclusion criteria focuses on patients diagnosed with heart failure during hospitalization. This selection criterion may overlook pre-hospitalization stages of HF. Despite this, Index diagnosis of HF through inpatient hospital admission constitutes over 80% [45] of all new HF diagnoses and may be associated with a significantly increased short-term risk of mortality and substantially higher long-term cost compared with community pathways [11]. Furthermore, selecting a prediction time exactly three years before a future heart failure hospitalization is a methodological choice that is only feasible in a retrospective study and does not reflect real-world scenarios where continuous prediction over different time points is required. Second, model validation was not performed on an out-of-sample dataset. This presents an urgent requirement to validate model generalisability in other healthcare systems outside of Scotland. This should be feasible due to routine availability of model features and is currently our primary focus of research. Third, the retrospective nature of this study resulted in a level of class balance that may not represent real-world prevalence. This phenomenon will affect the meaning of performance metrics such as Negative and Positive Predictive Values [46] and may be misleading when applied to different case-control ratios or other populations. Therefore, any future validation should involve prospectively-selected cohorts of patients and appropriately-selected validation metrics. Finally, despite our work on coefficient of determination, the black-box nature and the dimensionality of training data makes interpretation of our model unintuitive. This can present a challenge to clinical implementation.

In conclusion, this study demonstrates that a simple Transformer model utilising routinely-collected secondary care her data may offer a robust clinical decision support tool for community-based risk stratification of patients at risk of first HHF. Future work will involve a prospective study, which would allow for evaluation of the algorithm when exposed to real-world class distributions, assessing its effect on workflow safety and operational efficiency, including economic evaluation.
